# Synthetic Cannabinoid Agonist WIN 55212-2 Targets Proliferation, Angiogenesis, and Apoptosis *via* MAPK/AKT Signaling in Human Endometriotic Cell Lines and a Murine Model of Endometriosis

**DOI:** 10.3389/frph.2021.726936

**Published:** 2021-10-05

**Authors:** Harshavardhan Lingegowda, Jessica E. Miller, Ryan M. Marks, Lindsey K. Symons, Taylor Alward, Alan E. Lomax, Madhuri Koti, Chandrakant Tayade

**Affiliations:** ^1^Department of Biomedical and Molecular Sciences, Queen's University, Kingston, ON, Canada; ^2^Gastrointestinal Disease Research Unit (GIDRU), Queen's University, Kingston, ON, Canada; ^3^Department of Obstetrics and Gynecology, Kingston General Hospital, Kingston, ON, Canada; ^4^Division of Cancer Biology and Genetics, Queen's University, Kingston, ON, Canada

**Keywords:** cannabinoids, endometriosis, apoptosis, inflammation, angiogenesis

## Abstract

Endometriosis (EM) is characterized by the growth of endometrium-like tissue outside the uterus, leading to chronic inflammation and pelvic pain. Lesion proliferation, vascularization, and associated inflammation are the hallmark features of EM lesions. The legalization of recreational cannabinoids has garnered interest in the patient community and is contributing to a greater incidence of self medication; however, it remains unknown if cannabinoids possess marked disease-modifying properties. In this study, we assess the effects of synthetic cannabinoid, WIN 55212-2 (WIN 55), in EM-representative *in vitro* and *in vivo* syngeneic mouse models. WIN 55 reduced proliferation and angiogenesis *in vitro, via* MAPK/Akt-mediated apoptosis. These findings were corroborated in a mouse model of EM, where we found reduced TRPV1 expression in the dorsal root ganglia of the EM mouse model exposed to WIN 55, suggesting reduced signaling of pain stimuli. Ultimately, these pieces of evidence support the use of cannabinoid receptor agonists as a potential therapeutic intervention for EM associated pain and inflammation.

## Introduction

Endometriosis (EM) is a multifactorial, chronic inflammatory, and estrogen-dependent gynecological disorder characterized by the growth of endometrial tissue in extrauterine locations. EM affects 1 out of 10 women of the reproductive age worldwide, with over 10 million cases in North America alone (1, 2). Although the pathogenesis of EM is not entirely understood, Sampson's theory of retrograde menstruation is broadly accepted, whereby debris from menstrual shedding is refluxed back through the fallopian tubes and into the peritoneal cavity (3). EM is frequently associated with severe symptoms that include dysmenorrhea, vaginal hyperalgesia, chronic pelvic pain, and infertility (2, 4–6). Although EM is a global concern due to associated risks including ovarian cancer and other comorbidities, EM frequently goes undiagnosed and is often ineffectively treated due to limited therapeutic options. Management of severe pain among patients with EM is a particularly intractable medical challenge. Patients with EM are currently treated with nonsteroidal anti-inflammatory drugs for pain management, whereas hormonal contraceptives, GnRH antagonists and progestins, are recommended to partly manage lesion growth (5, 7, 8). However, this strategy of management is certainly not favorable for women who would like to conceive as long-term practices lead to the risk of side effects and a higher chance of recurrent lesion growth (7, 9). Despite significant advancements in the understanding of the disease, the clinical needs of patients with EM are yet to be managed with an optimal therapeutic regime, which is focused on synergistically controlling inflammation and pain among patients who are not responsive to traditional clinical strategies.

Cannabinoids have been studied extensively as a potential therapeutic in the field of cancer biology (10) and neurodegenerative diseases (11), which has resulted in identifying anti-inflammatory, anti-proliferative, and anti-analgesic properties. Humans have a complex endocannabinoid (EC) system that consists of EC ligands, such as 2-arachidonoylglycerol (2-AG) and *N*-arachidonoylethanolamine (AEA), classical receptors, such as cannabinoids receptor 1 (CB1) and 2 (CB2), and nonclassical receptors, such as G protein-coupled receptors (GPR) and transient receptor potential channels (TRPV) (12). CB1 is predominantly expressed in the nervous system and brain, modulating pain and neurotransmitter release, whereas, CB2 is highly expressed in the peripheral tissues including hepatic, reproductive, and lymphatic, reflecting various physiological processes (13). A growing body of evidence suggests that stimulation of CB1 and CB2 receptors in tumors inhibits proliferation and induces apoptosis *via* direct alteration of kinase pathway [PI3K/Akt, cyclic AMP-protein kinase-A (cAMP), ERK (extracellular signal-regulated kinase), and MAPK (mitogen-activated protein kinase)] (14). In addition, cannabinoids have been found to reduce hyperalgesia and neuropathic pain by calcineurin-dependent dephosphorylation of TRPV1 (15). These mechanisms suggest a potential intervention strategy in EM since proliferation, angiogenesis, and pain are the hallmarks of EM.

Due to the legalization of recreational cannabis in Canada, some parts of the US and selected European countries, it has garnered traction for self-management strategies among patients with EM to alleviate pain (16, 17). This combined with unsatisfactory treatment options has led some patients with EM to self-medicate with cannabis-based products (17, 18). Although clinical reports suggest cannabis can provide relief from pain in patients, we do not fully understand if or whether it alters the state of EM pathophysiology. In the current study, we, therefore, investigate the effects of non-selective, CB1/2 synthetic cannabinoid agonist WIN 55 in our EM microenvironment representative human cell lines, and a syngeneic mouse model of EM. Our findings based on *in vitro* studies show that WIN 55 inhibits proliferation, angiogenesis, and promotes apoptosis in a dose-dependent manner. We also provide evidence to suggest that cannabinoid receptor activation leads to alteration in MAPK and Akt signaling cascade, leading to alteration in cellular processes. Similarly, data from the mouse model of EM suggests that WIN 55 attenuates angiogenesis, proliferation and promotes apoptosis in EM-like lesions. Finally, we also provide critical evidence that WIN 55 affects TRPV1 expression in dorsal root ganglia (DRG) in the EM mouse model. Collectively, our findings suggest targeting cannabinoid receptors as one of the potential therapeutic opportunities for patients with EM in the near future.

## Materials and Methods

### Drugs and Solvent

WIN 55212-2 mesylate was purchased from Cayman chemicals (#10736 Michigan, USA) in the form of crystalline solid with ≥98% purity. WIN 55 has been extensively used in cancer research (19–21). Due to the chemical nature of WIN 55, solubility was a major concern and hence an emulsion-based solvent was used for both *in vitro* and *in vivo* experiments. Tocrisolve™ 100 (#1684 Bio-Techne, Canada) has been extensively used in both *in vitro* and *in vivo* studies (22, 23). WIN 55 stock solutions were prepared at 10 mM concentration in Tocrisolve™ 100 according to the protocol of the manufacturer and kept at 4°C until further use.

### Cell Culture

Immortalized human endometriotic epithelial cell lines (12Z), immortalized human endometrial stromal cells (HESC- T0533 ABM, Canada), and human umbilical vein endothelial cells (HUVECs- ATCC® CRL-1730™) were used in this study. 12Zs were grown in a T75 tissue culture flask as per previously described protocol (24) using DMEM/F-12 base media (11320033 ThermoFisher, Canada) supplemented with 5% fetal bovine serum (MP97068-085 VWR, Canada), 1% penicillin/streptomycin solution (15070063 ThermoFisher, Canada), and 1% sodium pyruvate (11360070 ThermoFisher, Canada). HESCs were grown in a Nunclon delta-treated T75 tissue culture flasks using Prigrow IV growth media (TM004 ABM, Canada) supplemented with 10% charcoal-stripped fetal bovine serum (12676029 ThermoFisher, Canada), 1% l-glumatine (A2916801 Thermofisher, Canada), and 1% penicillin/streptomycin solution. HUVECs were grown using a complete human EC growth medium (211–500 Cell Applications, USA) in a T75 flask incubated at 37°C and 5% CO_2_ until 90% confluent. Cell cultures were closely monitored to ensure cell morphology, and key properties (e.g., proliferation, phenotype) were unaltered.

### Cell Proliferation Assay

The 12Z and HESC cells were seeded at 0.5 × 10^4^ cells per well in an individual flat 96-well-plate using phenol red-free DMEM/F-12 (21041025 ThermoFisher, Canada) media and Prigrow IV media, respectively. Cells were incubated for 24 h before treating with vehicle (0.5% Tocrisolve™ 100), 1μM, 10 μM, 30 μM, and 50 μM WIN 55. Treated cells were incubated at 37°C and 5% CO_2_ for 22 h. The proliferation of cells was determined using WST-1 reagent (5015944001 Millipore Sigma, Canada). Ten microliters of WST-1 reagent were added to every 100 μl of supernatant and incubated for 2 h at 37°C and 5% CO_2_. The absorbance of formazan dye was recorded using SpectraMax iD3 microplate reader (Molecular devices, California, USA) at 450 nm wavelength with a reference wavelength reading at 650 nm. Results were calculated by subtracting background absorbance from blank media control.

### Apoptosis Assay

Immortalized human endometriotic epithelial cell lines, HESCs, and HUVECs were seeded individually at 0.5 × 10^4^ cells per well in a flat 96-well, clear bottom white-walled plate (3610 Corning, Canada) in their respective complete cell culture medium for 24 h. 12Zs, HESCs, and HUVECs were treated with vehicle (0.5% Tocrisolve™ 100), 1μM, 10 μM, 30 μM, and 50 μM WIN 55 for 22 h at 37°C and 5% CO_2_. Apoptosis in the culture was determined by Caspase-Glo® 3/7 assay system (G8091 Promega, Canada). A mixture of Caspase-Glo® buffer and Caspase-Glo® 3/7 substrate was added to the cell supernatant at a 1:1 ratio, homogenized at 300 RPM for 30 s, and incubated for 2 h at room temperature in a dark environment. Luminescence produced by luciferase was measured using a SpectraMax iD3 microplate reader (Molecular devices, California, USA). Results obtained were calculated by subtracting background luminescence from blank media control.

### Endothelial Tube Formation Assay

Human umbilical vein endothelial cells were utilized to perform endothelial tube formation as per the protocol of the manufacturer in a μ-slide assay format (#81506 Ibidi, Germany). Matrigel™ (#354230 Corning, USA) was used as the extracellular basement gel matrix for the endothelial cells. HUVECs were seeded into the μ-slide wells at 0.1 × 10^4^ cells per well in a 50 μl total volume of complete endothelial growth media and incubated for 30 min in a humid chamber at 37°C and 5% CO_2_. HUVECs were treated with vehicle (0.5% Tocrisolve™ 100), 1 μM, 10 μM, 30 μM, and 50 μM WIN 55 for 4 h in a humid chamber at 37°C and 5% CO_2_. Images were captured on Olympus CKX41 using a Lumenera Infinity 1–3 microscope camera. The entire frame of the images captured was submitted for tube formation analysis, which was unsupervised and carried out on the Wimasis image analysis platform (WimTube: Tube Formation Assay Image Analysis Solution) (25).

### Milliplex® Kinase Assay

The 12Z cells were treated with vehicle (0.5% Tocrisolve™ 100), 1 μM, 10 μM, 30 μM, and 50 μM WIN 55 for 24 h and were harvested and lysed using lysis buffer (#43-040 Millipore-Sigma, Canada). Millipex® kinase assay was carried out according to the protocol of the manufacturer. Briefly, lysed cells were filtered, and the protein concentration was evaluated using the Pierce™ microplate BCA protein assay kit (#23252 ThermoFisher, Canada). The cell lysate was normalized to 100 μg across the samples and 25 μg of total protein was used per sample, per well for the assay. Two panels of kinase assay were used in this study, namely, MAPK/SAPK signaling 10-plex (#48-660MAG Millipore-Sigma, Canada) and 2-Plex Total/Phospho Akt (#48-618MAG Millipore-Sigma, Canada). Beads containing analytes from both the kits were incubated with the protein lysate overnight at 4°C on a shaker. Samples were washed and incubated with a detection antibody tagged with Streptavidin-PE. Samples were subjected to the Luminex 200® system (Luminex®, USA) to acquire data, and the results were analyzed using Bio-Plex manager 6.2 (Bio-Rad, USA).

### Multiplex Cytokine Analysis of Cell Supernatant

Supernatant from 12Z, HESC, and HUVEC cells, treated with various concentrations of WIN 55, were collected. Cell supernatant was centrifuged to remove cells and debris. The supernatant was aliquoted as per requirements for multiplex cytokine analysis and stored at −80°C until further use. Cell supernatant was analyzed using commercially available 42 plex Human Cytokine array multiplex panel (HD42; Eve Technologies, Canada).

### RNA Extraction From 12Z and HUVEC Cells and Quantitative PCR

Total RNA from 12Z and HUVEC cells were extracted using a total RNA extraction kit (#37500 Norgen Biotek Corp, Canada) according to the protocol of the manufacturer. Total RNA obtained was subjected to reverse transcription to obtain cDNA template using first-strand cDNA synthesis kit (#330404 Qiagen, Canada). CB1, CB2, and GAPDH (reference) primers were designed using the NCBI primer tool. Quantitative PCR was performed using LightCycler 480 (Roche Diagnostics, Canada). The melting curve protocol was assessed to find out the presence of respective products or amplicons. A single melting peak represents the presence of a single product of interest amplified by the primers. CB1 forward (5′ GCCTTATTTCACAGTCTGATGGC 3′), CB1 reverse (5′ AAGGTGTGGTGGCCTTTTCT 3′), CB2 forward (5′ TGAAGATTGGCAGCGTGACT 3′), CB2 reverse (5′ CGGGTGAGCAGAGCTTTGTA 3′), GAPDH forward (5′ CATGTTCGTCATGGGGTGAACCA 3′), and GAPDH reverse (5′ AGTGATGGCATGGACTGTGGTCAT 3′).

### Animals

All surgical and non-surgical procedures were performed in compliance with the protocols approved by the Queen's University Institutional Animal Care Committee as per the guidelines provided by the Canadian Council of Animal Care. All animals were assigned randomly to the surgical procedures and treatment groups. Immunocompetent female C57Bl/6N mice (Charles Rivers, USA) were used for this study. Mice of 8–9 weeks old were used for all the experiments, and four to five mice were housed in a single cage with uninterrupted access to clean drinking water and food. Standard housing conditions were maintained throughout the study at 22 ± 1°C and 50 ± 10% humidity with a light cycle from 7 a.m. to 7 p.m. and dark cycle for the rest. All animals were acclimatized at the animal housing facility for 1 week before starting the experiments.

### Syngeneic Mouse Model of EM and WIN 55 Treatment

Endometriosis was surgically induced as described previously (26). Six independent groups were used for this time course study (*n* = 5–6). Briefly, the uterus from the donor C57BL/6N mice was harvested, and uterine horns were longitudinally dissected to reveal the endometrium. Uterine fragments were obtained using a 3.0 mm epidermal biopsy punch (Integra™ Miltex®, USA). Recipient mice were put under 3.5% isoflurane vaporizer anesthesia to make a midline incision in the abdomen (*n* = 5–6) and two 3.0 mm uterine fragments were implanted on the right inner peritoneal wall using a veterinary grade tissue bonding glue (3M Vetbond, 1469SB). Control groups (*n* = 5–6) were sham-operated with a midline incision in the abdomen without implantation of uterine fragments. Experimental groups designated to receive WIN 55 were injected at 1 mg/kg (i.p.) on alternative days. The dose of WIN 55 (1 mg/kg) was determined based on the evidence in literature across broad *in vivo* models such as systemic sclerosis and colitis (27, 28). Similarly, groups designated as controls received vehicle (1% Tocrisolve in PBS) on alternative days (i.p.). Experimental groups (*n* = 5–6) were euthanized on day 14 (mid) and 28 (late) after surgical induction of EM. Blood was harvested through submandibular bleeding. Peritoneal fluid was collected by injecting 3 ml of ice-cold phosphate buffered saline (PBS) into the peritoneal cavity. We harvested two endometriotic lesions per animal when possible (sometimes the lesion fuse together so one lesion in that case), harvested and processed using 4% paraformaldehyde overnight (12–20 h), kept at 4°C in 70% ethanol, and then embedded in paraffin for storage.

### Isolation of DRG

Mice induced with EM and treated with or without WIN 55 were decapitated after carbon dioxide asphyxiation to preserve the structural integrity of DRG. After euthanasia, the spinal column was isolated from T13 (thoracic level) to the S1 (sacral) region in a petri dish. Muscle and connective tissue were scraped off the vertebral column using a scalpel before removing the bone using bone ronguer (Blumenthal Rongeurs-15 cm, WPI, USA or similar). DRG from both sides of L1 to L5 was carefully removed using fine tip forceps (Dumont tweezers #5, WPI, USA). DRG was immediately transferred to ice-cold 4% paraformaldehyde for fixation over 8 h. DRG was embedded in paraffin block to construct a tissue microarray (TMA) with 1 mm core per well.

### Flow Cytometry

Harvested peritoneal fluid was passed through a 70 μm nylon cell strainer (#10054-456 Corning, Canada) to obtain a clump-free homogenous population. Peritoneal cells were counted on an automated cell counter (Countess 3, ThermoFisher), and 1 × 10^6^ cells were utilized for staining. Non-specific Fc receptors were blocked using 1 μg of TruStain FcX™ (anti-mouse CD16/32) antibody (#101319 Biolegend, USA) for 10 min on ice. Peritoneal cells were stained (antibodies purchased from Biolegend, USA, unless otherwise stated) according to the protocol of the manufacturer with APC tagged anti-mouse Ly-6C (#128016), PE/Cy7 tagged anti-mouse Ly-6G (#127618), PE/Dazzle 594 tagged anti-mouse CD19 (#115554), FITC tagged anti-mouse CD3 (#100204), APC/Cy7 tagged anti-mouse NK1.1 (#108724), AlexaFluor 700 tagged anti-mouse CD11b (#101222), and PE tagged anti-mouse F4/80 (#124801-80 ThermoFisher, Canada) for 20 min at 4°C. Stained cells were then washed using FACS buffer, followed by fixation of the cells using a 1:1 volume of intracellular fixation buffer (#88-8824-00 ThermoFisher, Canada). Cells were fixed for 30 min at 4°C before subjecting to wash using FACS buffer. A similar staining protocol was followed using isotype control and fluorochrome minus one (FMO) control to aid downstream analysis of the immune cell population. Stained and fixed cells were stored at 4°C overnight before analysis using Beckton Dickenson FACS Aria III (BD Biosciences, USA).

### Immunohistochemistry

Embedded lesions were sectioned at the 5 μm thickness and subjected to deparaffinization using xylene and rehydration through series of alcohol and citrosolve solution. Tissue sections were then subjected to immunostaining using an automated stainer (BenchMark XT, Ventana Medical System Inc, Tucson, USA) in the Department of Pathology and Molecular Medicine at Queen's University (Kingston, ON, Canada). Antigens were retrieved using Tris-EDTA solution and individual sections were then stained using primary polyclonal anti-CB1 (1:50, ThermoFisher, #PA1-743), anti-CB2 (1:50, ThermoFisher, # PA1-746A), anti-CD31 (1:100, NEB, # 77699S), anti-Ki67 (1:1000, Abcam, # ab15580), and anti-Caspase 3 (1:2000, NEB, #9662S). Further, sections were subjected to suitable secondary antibody staining, followed by chromogenic development of stain using Ultrablue DAB detection kit (Ventana medical systems Inc, Tucson, AZ, USA). Finally, sections were stained with nuclear stain (hematoxylin) and blueing agent, followed by the addition of coverslip prior to scanning. Slides were scanned using Olympus VS120 Virtual Slide Microscope (Olympus, USA) and images were analyzed on Halo® image analysis platform (Indica labs, USA). An entire section of the lesions was subjected to image analysis using singular computer-generated algorithms (unaltered between different lesions) created for each Ki67, Caspase-3, CD-31, CB-1, and CB-2 with more than one observer.

### Immunofluorescence

TMA of DRG was sectioned at 5 μm thickness and subjected to deparaffinization as described above. TMA section was stained using BOND RX (Leica biosystems, USA) stainer with manual addition of primary, secondary antibody, and DAPI nuclear stain. Citrate solution at pH 6 was used for antigen retrieval with an incubation time of 40 min. Sections were stained with polyclonal anti-TRPV1 primary antibody (1:250, ThermoFisher, #PA5-77317) for 10 min at room temperature in a humid chamber. Excess antibody was washed off and Donkey anti-Rabbit polyclonal antibody conjugated with Alexa Fluor 488 (1:1000, ThermoFisher, #R37118) was applied onto the sections and incubated for 8 min at room temperature in a humid chamber. ProLong™ Gold Antifade Mountant with DAPI (ThermoFisher, #P36931) was used for nuclear staining and mounting. Immunolabeled TMA sections were scanned using Olympus VS120 Virtual Slide Microscope (Olympus, USA) and images were analyzed on Halo® image analysis platform (Indica labs, USA).

### Statistical Analysis

Statistical analysis was conducted using GraphPad Prism 9.0 (GraphPad, USA). Data are expressed as mean ± SD. Data obtained from proliferation, apoptosis, angiogenesis, and cytokine analysis of 12Z, HESC, and HUVEC cells, and supernatant were analyzed using ordinary one-way ANOVA with Tukey *post-hoc*. Data obtained from IHC analysis of lesion sections were analyzed using a non-parametric unpaired student's *t*-test. Data from IHC and immunofluorescence of DRG, as well as the flow cytometry data from *in vivo* experiments, were subjected to ordinary two-way ANOVA with Sidak *post-hoc*. Values of *p* < 0.05 were considered statistically significant.

## Results

### WIN 55 Attenuates Proliferation of Human Endometriotic Epithelial and Endometrial Stromal Cells *in vitro* via Apoptosis

**Figure 1:**
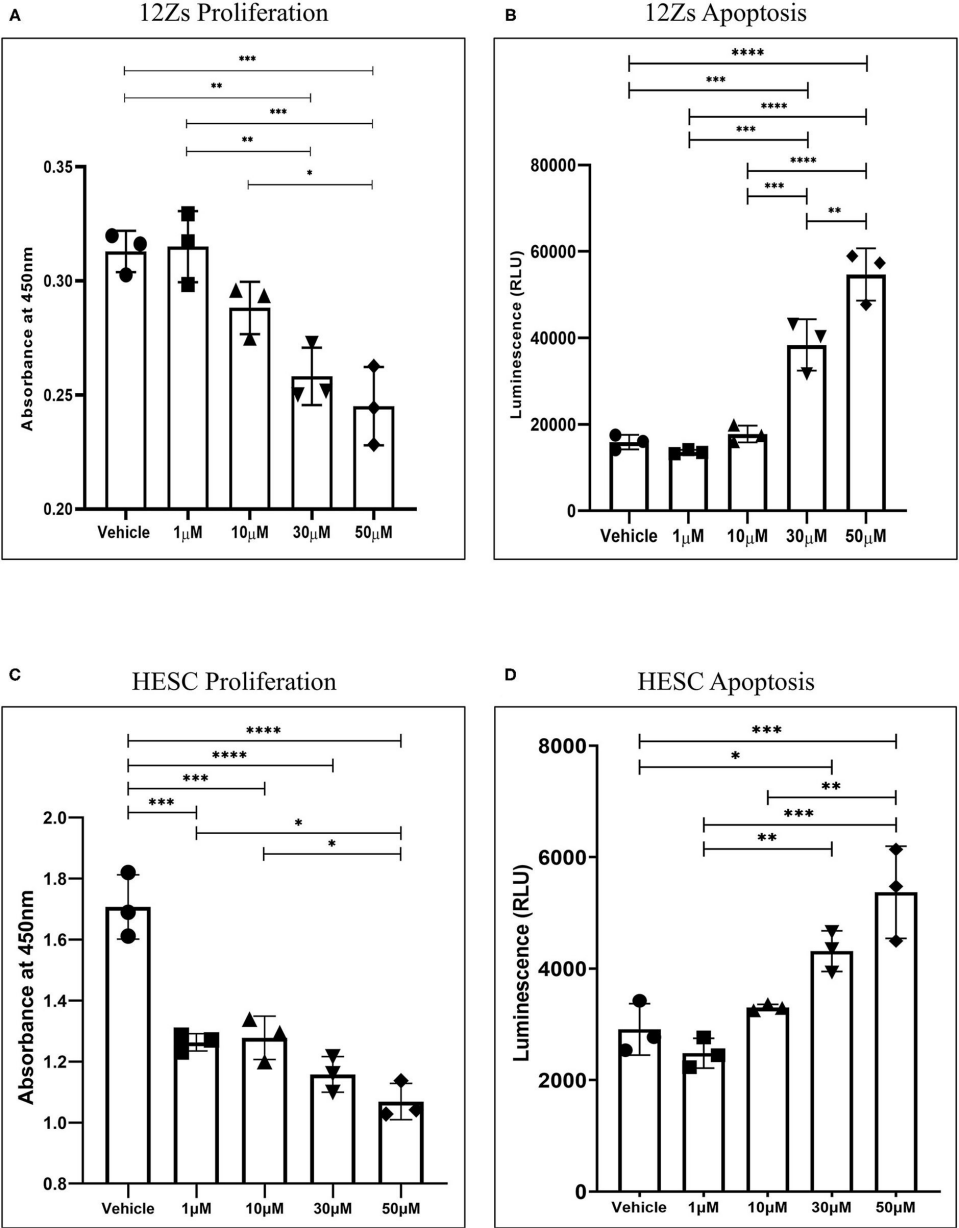
Stimulation of CB1 and CB2 receptors in endometriotic epithelial cells (12Z) and endometrial stromal cells (HESC) attenuates proliferation by triggering apoptosis. **(A)** Treatment with various concentrations of WIN 55 leads to attenuation of proliferation in a dose-dependent manner. **(B)** Caspase 3/7 activity in the cell supernatant showed that WIN 55 exposure triggered apoptosis in 12Z cells. **(C)** WIN 55 exposure in HESC cells showed concentration-dependent reduction in proliferation with WIN 55 concentration as low as 1 μM. **(D)** Apoptosis in HESC cells exposed to WIN 55 showed a similar dose-dependent increase in caspase 3/7 activity as observed in 12Z cells. Ordinary one-way ANOVA with Tukey *post-hoc*. *P < 0.05 **P < 0.01 ***P < 0.001 ****P < 0.0001.

Our first aim was to identify the effects of WIN 55 on proliferation and apoptosis in endometriotic epithelial (12Z) (24) and endometrial stromal (HESC) cell lines. Both CB1 and CB2 receptors were expressed in 12Z cells as confirmed with qPCR analysis (Supplementary Figures 1A,B). The EC_50_ value of WIN 55 has been reported to range from ~105.5nM to 26μM, depending on the cell type (29–31). However, the EC_50_ value of WIN 55 has not been determined in either 12Zs, HESCs, or HUVECs. Hence, a wide range of WIN 55 concentrations have been used based on estimated EC_50_ and previous publications in EM and cancer biology (32, 33). 12Z and HESC cells exposed to WIN 55 at varying concentrations (1, 10, 30, and 50 μM) were assessed for proliferation using WST-1 assay. We observed that WIN 55 significantly reduced the proliferation of 12Z cells (Figure 1A) and HESCs (Figure 1C) in a concentration-dependent manner. To identify the potential mechanism of action, we further investigated the effects of WIN 55 on apoptosis in 12Z and HESC cells. Caspase 3/7 enzymatic activity revealed that WIN 55 induced significant apoptosis in a concentration-dependent manner in both 12Z (Figure 1B) and HESCs (Figure 1D). Some of the cytokines produced by 12Z and HESC cells upon exposure to WIN 55 were measured using multiplex cytokine assay (Supplementary Figures 2A–H).

### WIN 55 Exposure Inhibits Tube Formation Property of HUVECs

**Figure 2:**
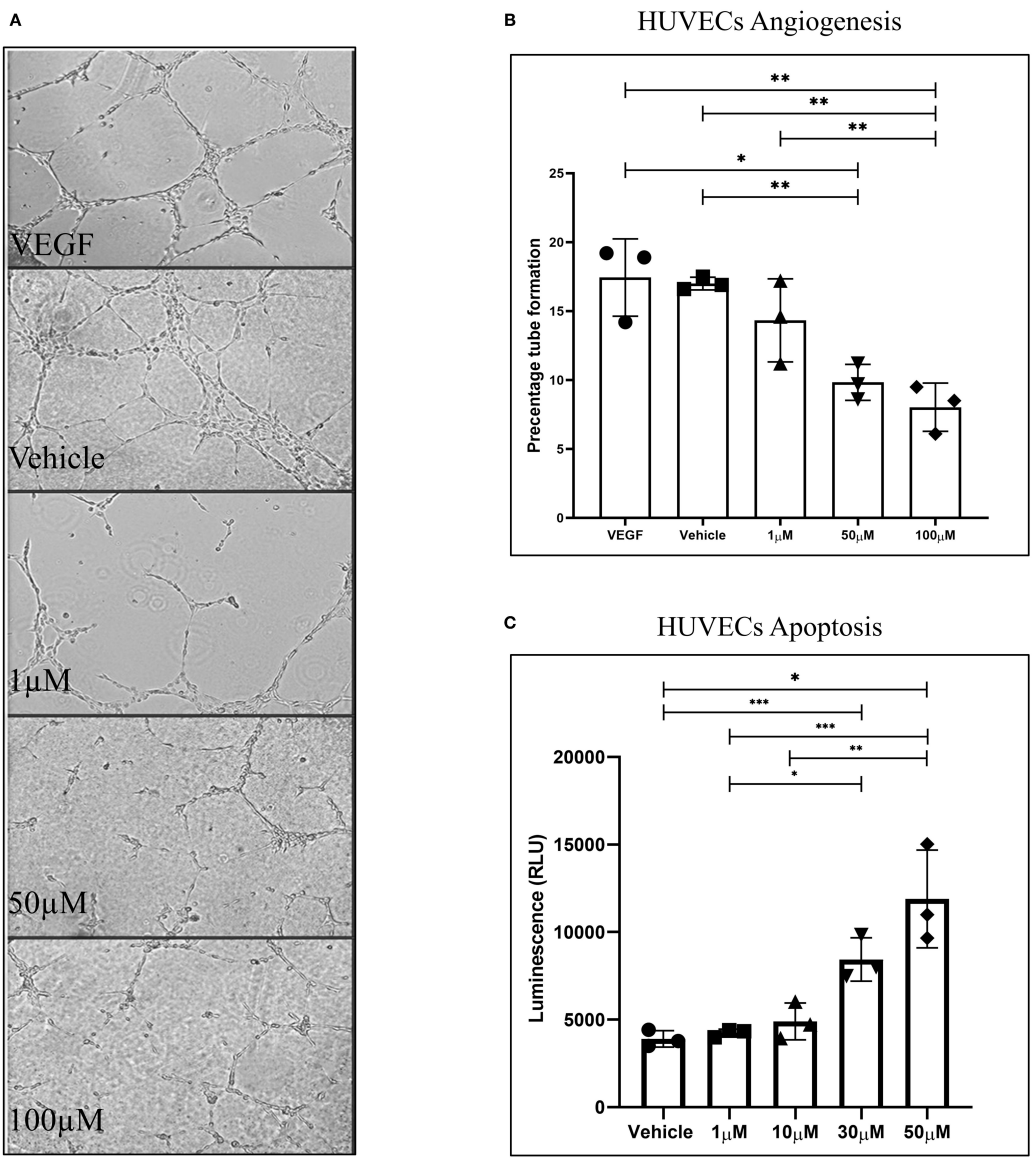
WIN 55 exposure inhibits tube formation ability in human umbilical vein endothelial cells (HUVECs). **(A)** Images captured shows alteration of tube formation in concentrations as low as 1 μM and near complete reduction in high doses. **(B)** The percentage of tube formation analysis showed a statistical significance across the board with a dose-dependent behavior. **(C)** Treatment with various doses of WIN 55 leads to dose-dependent increase in caspase activity in HUVECs. Ordinary one-way ANOVA with Tukey *post-hoc*. *P < 0.05 **P < 0.01 ***P < 0.001.

In this study, we assessed the ability of WIN 55 to affect angiogenesis and tube formation of HUVECs. CB1 and CB2 receptors were expressed in HUVECs as confirmed with qPCR analysis (Supplementary Figures 1C,D). HUVECs were allowed to propagate on Matrigel® and later exposed to low (1 μM), medium (50 μM), and high dose (100 μM) of WIN 55. Disruption of tube formation was found in WIN 55 dose as low as 1 μM (Figure 2A), and significant dose-dependent disruption of tube formation was observed in 50 and 100 μM concentrations (Figure 2B). To further establish whether WIN 55 causes apoptosis in HUVECs, we subjected HUVECs to Caspase 3/7 enzymatic activity through luciferase-aided luminescence. Analogous to 12Z cells, HUVECs also showed a concentration-dependent increase in apoptosis in response to WIN 55 treatment (Figure 2C). Cytokines produced by HUVECs in response to WIN 55 treatment were analyzed using multiplex cytokine analysis (Supplementary Figures 2I–L).

### Activation of CB1 and CB2 Receptors Alters the Signaling Cascade of MAPK Associated Kinase and Total Akt

**Figure 3:**
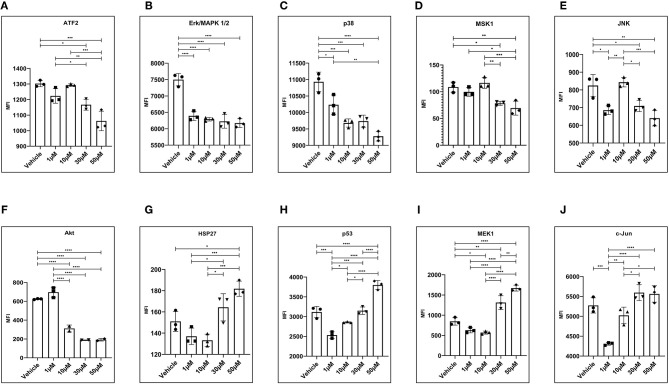
MAPK and Akt protein kinases are significantly altered in 12Z cells treated with WIN 55. **(A-F)** Total protein obtained from 12Z cells treated with WIN 55 showed significantly lower levels of ATF2, Erk 1/2, p38, MSK1, JNK, and Akt. **(G-I)** On the other hand, protein levels of HSP27, p53, and MEK1 were significantly higher in the cells treated with WIN 55. **(J)** Although a positive trend was noticed, c-Jun levels did not show significant changes when compared with cells treated with vehicle, apart from 1 μM concentration. Ordinary one-way ANOVA with Tukey *post-hoc*. *P < 0.05 **P < 0.01 ***P < 0.001 ****P < 0.0001.

We have evaluated the activity of MAPK family kinases and Akt in 12Z cells exposed to WIN 55 using a multiplex kinase assay kit. MAPK family proteins such as ATF2 (Figure 3A), Erk 1/2 (Figure 3B), p38 (Figure 3C), MSK1 (Figure 3D), and JNK (Figure 3E) showed a significant reduction in the mean fluorescence intensity, suggesting reduced levels in 12Z cells exposed to various doses of WIN 55 compared with a vehicle. Whereas proteins involved in signal relay to cells and back, such as HSP27 (Figure 3G), p53 (Figure 3H), and MEK1, (Figure 3I) were found to be significantly higher in the 12Z cells treated with WIN 55 compared to a vehicle. In addition, total Akt in the 12Z cells treated with WIN 55 also showed a significant reduction in MFI when compared with vehicle (Figure 3F). Although a positive trend was observed, no significant differences in MFI of c-JUN analytes were observed, except 1 μM concentration, in WIN55-treated cells compared with vehicle (Figure 3J).

### The Intervention of WIN 55 in a Syngeneic Mouse Model of EM Affects Lesion Growth and Survival

**Figure 4:**
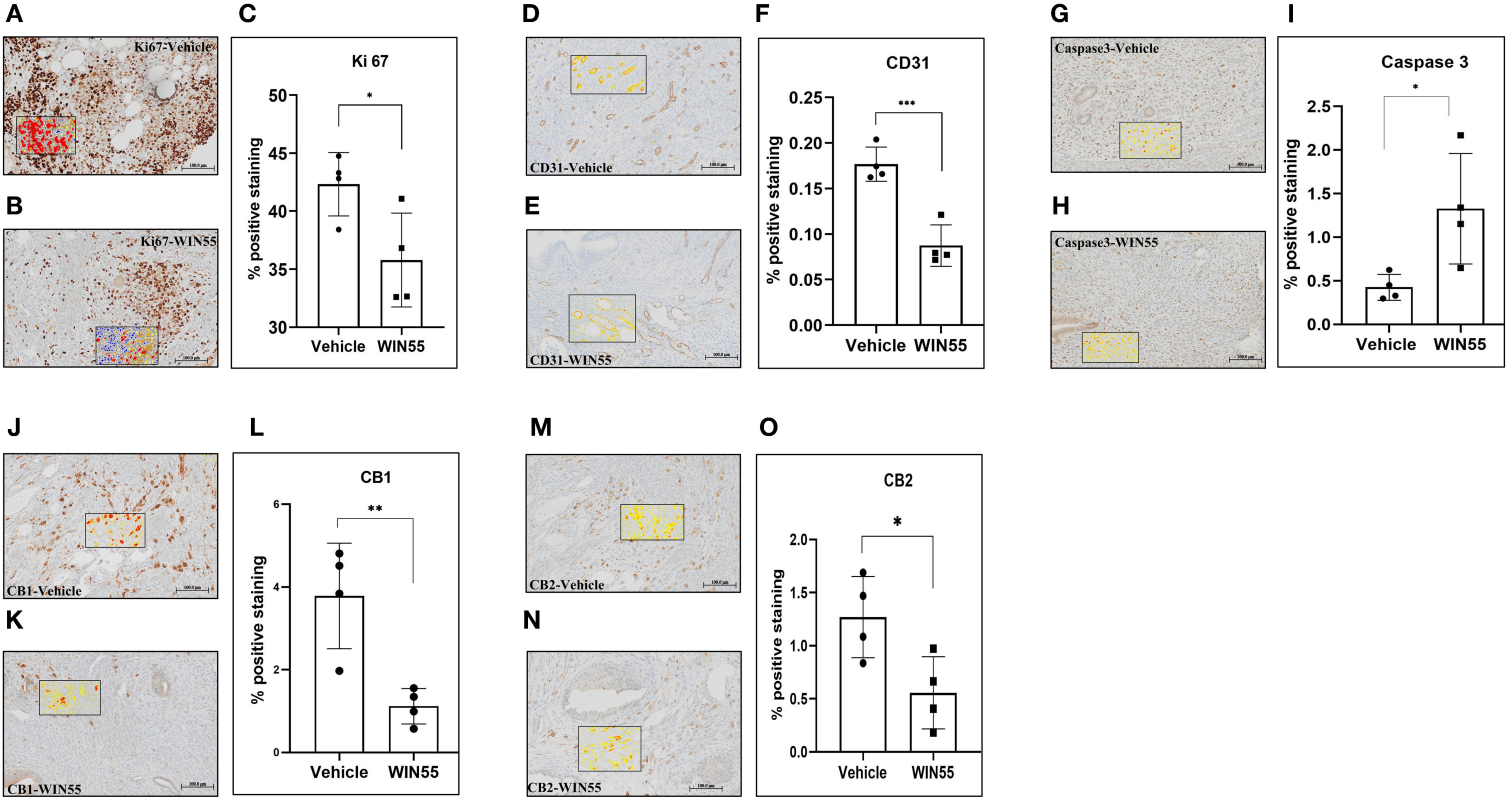
Chronic exposure of WIN 55 in endometriosis (EM) induced mice attenuates lesion growth and vascularization. Immunocompetent C57Bl/6N mice were induced with EM and treated with 14 doses of WIN 55 (1mg/kg i.p) before euthanasia on day 28. Lesions recovered from vehicle, and WIN 55 treated mice were analyzed for hallmarks of EM, such as proliferation and angiogenesis. Representative IHC images of Ki-67 **(A,B)** (marker of proliferation), CD31 **(D,E)** (marker of vasculature), Caspase-3 **(G,H)** (marker of apoptosis), CB1 **(J,K)**, and CB2 **(M,N)**. Lesions from mice that were subjected to chronic exposure of WIN 55 showed a significant reduction in tissue proliferation **(C)** and angiogenesis **(F)**, while caspase activity was significantly increased **(I)**, which indicates heightened apoptosis. Cannabinoid receptors CB1 **(L)** and CB2 **(O)** also showed reduced expression in these lesions compared with the group treated with vehicle. The black box within the IHC images represents the analysis used in this study, where yellow, orange, and red denotes weak, mild, and strong staining, respectively. Scale bar 100 μm. Non-parametric unpaired student's *t*-test. *P < 0.05 **P < 0.01 ***P < 0.001.

To determine whether WIN 55 would impact the local lesion microenvironment, we conducted *in vivo* studies using our well-established syngeneic mouse model of EM (26). Mice induced with EM were treated with or without WIN 55 for 28 days (1 mg/kg intraperitoneal (i.p) injection on alternate days) after the induction of EM. Immunohistochemical analysis revealed significant changes in the lesion histology that indicates the extent of EM lesion development. Endometriosis-like lesions from mice treated with WIN 55 showed a significant reduction in Ki-67 staining, a marker of proliferation, compared to lesions from vehicle-treated mice (Figures 4A–C). We also observed a significant reduction in CD31 staining (a marker of vasculature) in the lesions from mice treated with WIN 55 compared with mice treated with vehicle, indicating the effect of WIN 55 on angiogenesis (Figures 4D–F). Furthermore, endometriotic-like lesions from mice treated with WIN 55 showed significantly increased Caspase-3 staining which correlates to increased apoptosis, compared to mice treated with vehicle (Figures 4G–I). Finally, relevant to the biology of EC signaling, we found that CB1 (Figures 4J–L) and CB2 (Figures 4M–O) receptor staining was significantly reduced in the lesions from mice treated with WIN 55, compared with mice treated with vehicle. These results were also recapitulated in our 14-day experimental groups (*n* = 5/group), with the exception of Ki67 staining, which showed no significant differences (Supplementary Figure 3). Analysis of peritoneal immune cell populations on days 14 and 28 did not show any significant differences in general immune cell types categorized as the following: T cells (CD3), myeloid cells (CD11b), B cells (CD19), granulocytes (Ly6C and Ly6G), and macrophages/eosinophils (F4/80) (Supplementary Figures 4A–F). In summary, we show that WIN 55 treatment in a syngeneic mouse model of EM has direct and selective effects on lesion characteristics, such as proliferation, angiogenesis, and apoptosis.

### WIN 55 Exposure Alters TRPV1 Expression in DRG in a Syngeneic Mouse Model of EM

**Figure 5:**
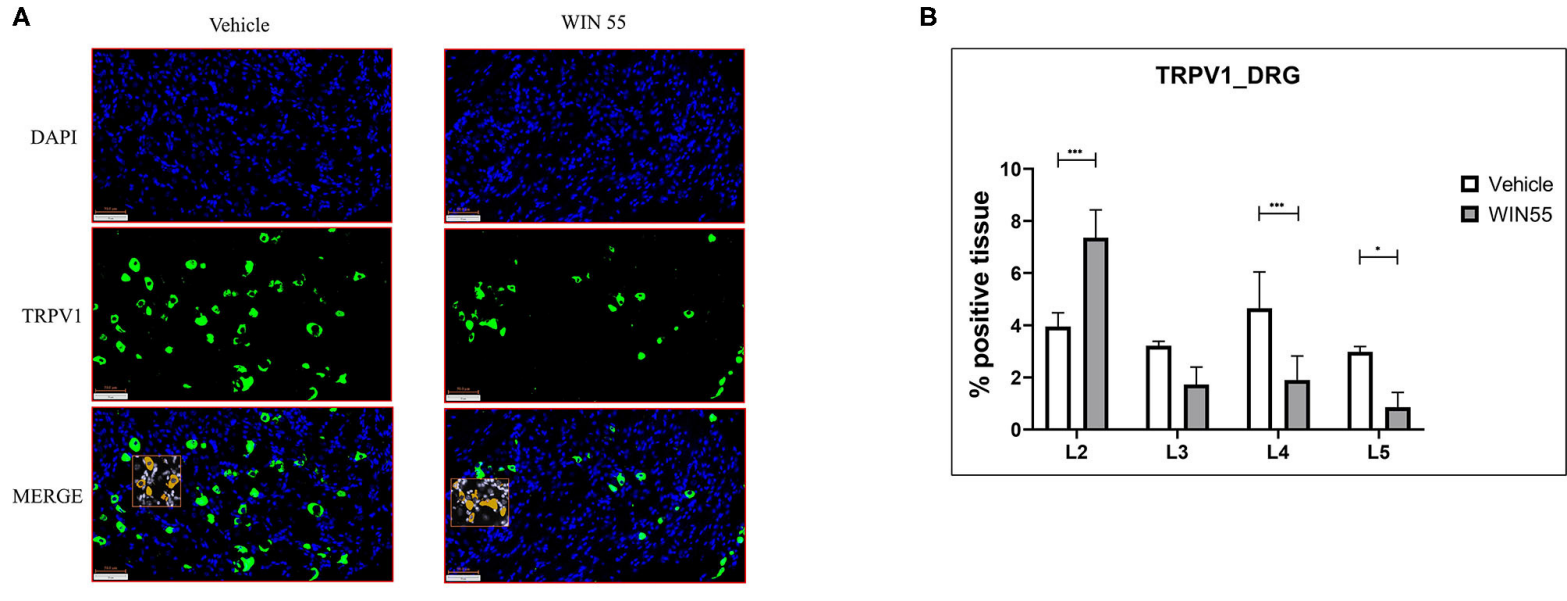
Cannabinoid receptor stimulation alters TRPV1 expression in the dorsal root ganglia of mice with endometriosis. Lumbar region DRG from L1 to L5 were analyzed for TRPV1 expression through immunofluorescence. **(A)** Representative images of blue (DAPI positive) and green (TRPV1 positive) channel and merged (DAPI and TRPV1 positive) layers show TRPV1 expression in DRG isolated from both vehicle and WIN 55 treated animals. **(B)** Statistical analysis of TRPV1 expression showed increased abundance in L2 of DRG isolated from WIN 55 treated mice, whereas L4 and L5 DRG showed significantly reduced expression when compared with mice treated with vehicle. The orange box within the immunofluorescence images represents the analysis used in this study. Scale bar 100 μm. Ordinary two-way ANOVA with Sidak *post-hoc*. *P < 0.05 ***P < 0.001.

In this study, we sought to identify if WIN 55 has any effects on TRPV1 receptor expression in DRG neurons, a subset of which is responsible for the detection and signaling of painful stimuli to the central nervous system (Figures 5A,B). Lumbar region L4 and L5 of DRG showed significantly decreased TRPV1 staining in mice treated with WIN 55 compared with mice treated with vehicle (Figure 5B). Although not significant, L3 DRG showed reduced TRPV1 staining in WIN 55-treated mice. However, L2 DRG from mice treated with WIN 55 showed a significant increase in TRPV1 staining compared to mice treated with vehicle (Figure 5B). In addition, we evaluated CB1 receptor expression in the DRG and found no significant differences between mice treated with or without WIN 55 (Supplementary Figures 5A,B). Together, we show that WIN 55 exposure alters the TRPV1 expression in DRG of mice induced with EM and might contribute to reduced hyperalgesia in these mice.

## Discussion

In this study, we demonstrated that the synthetic cannabinoid, WIN 55, inhibits hallmark features of EM, such as angiogenesis in HUVECs and proliferation in HESC and 12Z cells by targeting MAPK and Akt signaling cascade. The *in vitro* proliferation assays demonstrated that activation of cannabinoid receptors using WIN 55 inhibits both HESC and 12Z cell growth by driving apoptosis. These findings further strengthen the original observations of Leconte et. al, where they documented the anti-proliferative effects of WIN 55 in endometriotic stromal cells from patients with severe EM (32). The decrease in endometriotic stromal cell proliferation was associated with inhibition of AKT activation (32). Moreover, the anti-proliferative properties of WIN 55 have been explored in a number of cancer therapeutic studies [discussed in detail here (34)]. However, WIN 55 is a non-selective agonist of CB1 and CB2 receptors; further investigation is necessary to understand CB1 and CB2 specific responses by knockout models and/or receptor specific agonists (35). Vascularization is a key biological process that aids in the proliferation of EM lesions by a constant supply of oxygen and nutrients through the blood. Initiation of EM is believed to be due to retrograde menstruation, and adhesion of endometrial debris, and the refluxed tissue in patients with EM has decreased apoptosis compared to healthy fertile women, which could contribute to the lack of clearance (36). Although the mechanism is not entirely known, studies in cancer cell lines have suggested that stimulation of cells with WIN 55 induces cell cycle arrest, eventually leading to apoptosis (33, 37). Even though evidence suggests a potential mechanism of action through modulation of immune cells and cytokines, there is a discrepancy in recapitulating these effects (38). We aimed to identify if WIN 55 exposure affects the cytokines produced by 12Z and HUVECs. We did not find any significant changes in the levels of key cytokines, such as vascular endothelial growth factor (VEGF). The lack of cytokine production in these cell lines in response to WIN 55 stimulation may be due to the absence of an inflammatory milieu. Regardless, these data provide a foundation and show that stimulation of cannabinoid receptors inhibits key pathophysiological processes in EM, such as proliferation and angiogenesis.

Previous studies have mapped the kinase activity that is detrimental to inflammation, proliferation, and sustained growth of endometriotic lesions [as discussed here (39)], of which, the MAPK and PI3K/Akt pathways are crucial in determining the survivability of cells under stress. Various studies have independently concluded that the protein kinases, such as p38 (MAPK) and Akt, where both induce a pro-inflammatory response, secretion of growth factors, and proliferation of cells, were found to be highly active in the endometriotic cells from women with EM (40–42). Our study assessed the MAPK family kinases and total Akt in 12Z cells, where protein kinases such as p38, Erk 1/2, Akt, JNK, ATF2, and MSK1 were found to be significantly reduced, whereas the kinases that are involved in signal relays, such as p53, HSP27, and MEK1, were significantly increased, suggesting a positive feedback loop with the transcription factors. Our study is partially contrary to studies conducted by Leconte et. al. where they concluded that WIN 55 treatment did not produce profound effects on Erk 1/2 signaling in primary endometriotic stromal cells. Although the cell types were different, we found that WIN 55 exposure significantly reduced the Erk 1/2 activation in the endometriotic epithelial cell line (12Z). However, our studies are in line with the argument that WIN 55 exposure had significant effects on the activation of the Akt pathway (32). On the other hand, studies in pancreatic cancer cells have shown that activation of cannabinoid receptors resulted in p38 pathway alteration, which reduced inflammation and improved the condition in mice (23). In fact, pancreatic cancer cells treated with cannabinoids induce autophagy-mediated cell death through the Akt-mTORC signaling axis (43), whereas p53, in association with heat shock proteins, has been found to induce caspase-independent apoptosis in glioblastoma cells (44). Although this approach seems plausible as a treatment for EM, further studies are necessary to validate the pathway through targeted inhibition and/or activation, as these protein kinases are also an integral part of decidualization, implantation, and normal homeostasis (45).

WIN 55 treatment in our syngeneic mouse model revealed that it not only impacted lesion proliferation but also induced apoptosis and halted angiogenesis. Even though we did not observe changes in immune cell profiles and cytokines in the peritoneal microenvironment, WIN 55 targets lesion-associated proliferation and vascularization. However, further validations are necessary to assert the selective function of WIN 55. These observations are in accordance with the known functions of WIN 55 in other conditions, including neurodegenerative disorders and cancer. It would be prudent to establish whether and how EC components are dysregulated in the EM lesion microenvironment. Based on these, we will be able to broadly target both CB1 and CB2 receptors using selective agonists. Studies by Leconte et. al, have explored the avenue of WIN 55 exposure in nude mice induced with EM. Although not comprehensive, they found significantly reduced lesion volume between WIN 55 treated and untreated groups (32). Our study provides evidence that the WIN 55-induced effects on the endometriotic-like lesions are multifaceted and target several processes. Results from our *in vitro* experiments were corroborated in our mice model where we found significantly reduced proliferation and vascularization, whereas apoptosis was significantly higher in the lesions from mice treated with WIN 55 when compared with lesions from mice treated with vehicle. Indeed, the complimentary findings of a broad-spectrum multiplex kinase analysis in 12Zs (human endometriotic epithelial cells) treated with varying concentrations of WIN 55 support the molecular and cellular kinase pathways targeted by activation of CB1 and CB2 receptors.

Pain is one of the most reported and common clinical features associated with EM. Patients with EM experience varied pain, which is often chronic in nature, due to a number of mechanisms (nociceptive, inflammatory, and neuropathic). Previous reports have indicated that neuroangiogenesis drives the pain, whereas factors such as inflammation and hormone imbalance promote hypersensitivity [as discussed here (46)]. Pain is subjective in assessment and reporting, and hence, we opted to objectively assess the activity of TRPV1 in the DRG of mice with or without exposure to WIN 55. Regardless of the degree of pain experienced, the peripheral and central nervous systems are involved in transduction, transmission, modulation, and perception of pain (46). A recent study using a rat model of EM showed that the TRPV1 expression in DRG was significantly higher and suggested that it may sensitize the peripheral nervous system, which in turn contributes to EM-associated pain (47). In addition to cannabinoid agonists possessing anti-proliferative and anti-angiogenic properties, studies have identified that activation of cannabinoid receptors leads to reduced neural inflammation, potentially through direct dephosphorylation of TRPV1 (48, 49). In line with these studies, we show that stimulation of classical and nonclassical receptors using WIN 55 targets the expression of the TRPV1 channel in the DRG of an EM mouse model. It is important to note that although TRPV1 is necessary for inflammatory hypersensitivity, its expression and function are target tissue dependent. Although a positive avenue for therapeutic approach for pain in patients with EM, cannabinoid signaling is a complex architecture, and hence, the mechanism of action is yet to be fully understood.

**Figure 6:**
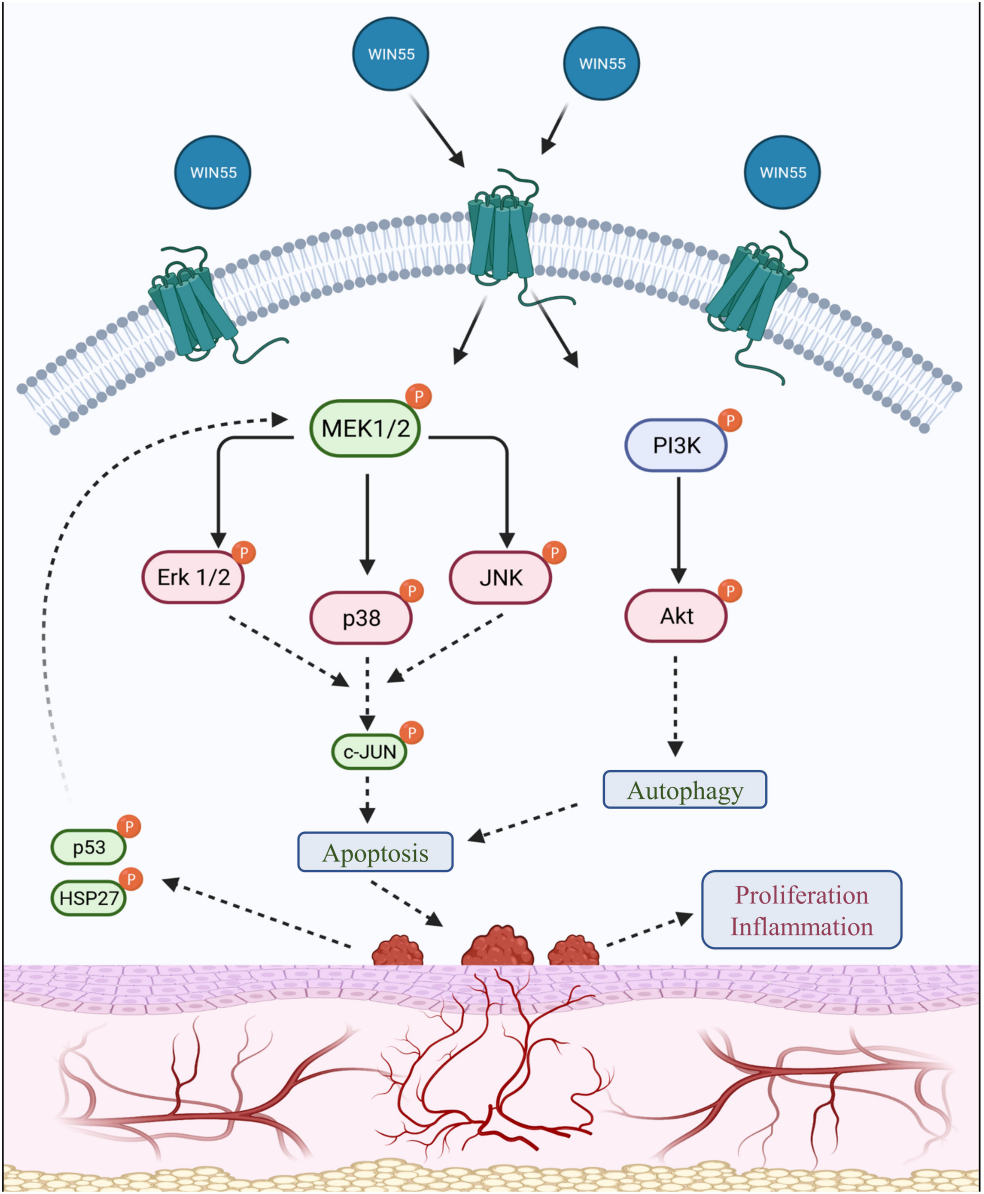
Summary of the signaling cascade triggered by cannabinoid receptor activation in endometriosis. A summary of the downstream signal alteration upon cannabinoid receptor activation is outlined in this schematic representation. Protein kinases, such as MAPK (p38) and PI3K/Akt family members, are involved in cell growth, migration, and survival under normal circumstances. Endometrial tissues have been identified to possess higher activity of kinases such as MAPK and Akt, which contribute to increased proliferation. Protein levels assessed in this study show that CB1 and CB2 receptor activation by WIN 55 leads to downstream modulation of important MAPK family proteins, such as Erk 1/2, p38, JNK, c-JUN, p53 and HSP27, as well as PI3K/Akt axis to trigger autophagy and eventually apoptosis through caspase-dependent and/or independent pathway. The use of red and green colors within the labeling represents downregulation and upregulation, respectively.

In conclusion, we show that WIN 55 exposure *in vitro* and *in vivo* alters the hallmarks of EM, such as proliferation and angiogenesis, and drives apoptosis by altering the protein kinase signaling cascade (Figure 6). Most importantly, stimulation of cannabinoid receptors through WIN 55-reduced TRPV1 expression in the DRG of an EM mouse model, which could affect the perception of pain. Although our study shows positive outcomes, we cannot conclude the mechanism of cannabinoid receptor activation in EM, which may be attributed to the complex nature of both EM and the EC network. Cannabinoids are also at a higher potential for abuse and side effects (50). Limitations also extend to the animal model of EM with fundamental differences between mice and humans, such as a lack of murine menstruation, differing hormone levels, lack of chronic inflammation, and increased sensitivity to pain. Nonetheless, in line with several articles, cannabinoids have the potential to target the hallmarks of EM. Further studies are necessary to understand the underlying mechanism of action of cannabinoids in EM while mitigating side effects.

## Data Availability Statement

The original contributions presented in the study are included in the article/Supplementary Material, further inquiries can be directed to the corresponding author/s.

## Ethics Statement

The animal study was reviewed and approved by University Animal Care Committee Queen's University, Kingston, ON, Canada.

## Author Contributions

HL and CT conceived the experiments. CT and MK contributed reagents, supervised data analysis, and provided funding. HL conducted the majority of the experiments, analyzed all results, and wrote the manuscript. JM, RM, TA, and AL helped with experiments and data analysis. All the authors reviewed the manuscript.

## Funding

This research was supported with funds from the Canadian Institutes of Health Research (CIHR-394340, MK, and CT).

## Conflict of Interest

The authors declare that the research was conducted in the absence of any commercial or financial relationships that could be construed as a potential conflict of interest.

## Publisher's Note

All claims expressed in this article are solely those of the authors and do not necessarily represent those of their affiliated organizations, or those of the publisher, the editors and the reviewers. Any product that may be evaluated in this article, or claim that may be made by its manufacturer, is not guaranteed or endorsed by the publisher.

## Supplementary Material

Supplementary Figure 1 Expression of cannabinoid receptors (CB1 and CB2) in 12Zs and HUVECs. **(A–D)** Untreated 12Z and HUVECs were utilized to extract RNA and perform qPCR to evaluate the presence of CB1 and CB2 receptor expression. Double lines represent duplicates, whereas the peak generated for the melting curve shows the presence of a single product being amplified by the primers.

Supplementary Figure 2 Cytokine production in 12Zs, HESCs, and HUVECs under various doses of WIN 55. Cytokines produced by the cells were analyzed using a multiplex cytokine analysis. **(A–L)** Some of the cytokines produced by 12Zs, HESCs, and HUVECs include CCL2, GM-CSF, EGF, PDGF-AA, IL-4, IL-8, and VEGF-A. Ordinary one-way ANOVA with Tukey *post-hoc*. ^*^*P* < 0.05 ^**^*P* < 0.01 ^***^*P* < 0.001 ^****^*P* < 0.0001.

Supplementary Figure 3 WIN 55 exposure for 14 days in mice model of endometriosis shows changes similar to 28 days. Representative IHC images of Ki-67 **(A,B)** showed no changes in percentage positive staining **(C)** upon analysis. However, CD31 **(D,E)**, CB1 **(G,H)**, and CB2 **(J,K)** showed a significant reduction in percentage positive staining (**F,I,L** respectively). The red box within the images represents the analysis used in this study, where yellow, orange, and red denotes weak, mild, and strong staining, respectively. Yellow or weak staining has been omitted from the analysis as background noise. Nonparametric unpaired student's *t*-test. ^*^*P* < 0.05. Scale bar 50μm.

Supplementary Figure 4 Flow cytometry analysis of immune cell population from 14 and 28 days endpoint. **(A–F)** Relative abundance of peritoneal immune cell population includes CD3, CD11b, CD19, Ly6C, Ly6G, and F4/80. Two-way ANOVA with Sidak *post-hoc*.

Supplementary Figure 5 Expression of CB2 in the lumbar DRG of mice and time associated expression of CB2 in endometriosis-like lesions from mice. **(A–B)** CB1 expression was determined immunohistochemically in the lumbar DRG of mice treated with WIN 55, which did not reveal any significant difference. Finally, we induced endometriosis and sacrificed mice at different time points to assess CB2 expression. **(C)** Although not significant, we saw time-dependent increase in CB2 expression in the lesions. Two-way ANOVA with Sidak *post-hoc* **(B)**. Ordinary one-way ANOVA with Tukey *post-hoc* **(C)**.
