# Supplementary material for: Synthetic Cannabinoid Agonist WIN 55212-2 Targets Proliferation, Angiogenesis, and Apoptosis via MAPK/AKT Signaling in Human Endometriotic Cell Lines and a Murine Model of Endometriosis
Source: Front Reprod Health. 2021 Oct 5;3:726936. doi: 10.3389/frph.2021.726936 (PMC9580784; doi:10.3389/frph.2021.726936)
Supplement: Supplementary Figure 1 — Expression of cannabinoid receptors (CB1 and CB2) in 12Zs and HUVECs. (A–D) Untreated 12Z and HUVECs were utilized to extract RNA and perform qPCR to evaluate the presence of CB1 and CB2 receptor expression. Double lines represent duplicates, whereas the peak generated for the melting curve shows the presence of a single product being amplified by the primers. [file Data_Sheet_1.docx]

**Supplementary Figure 1**

**
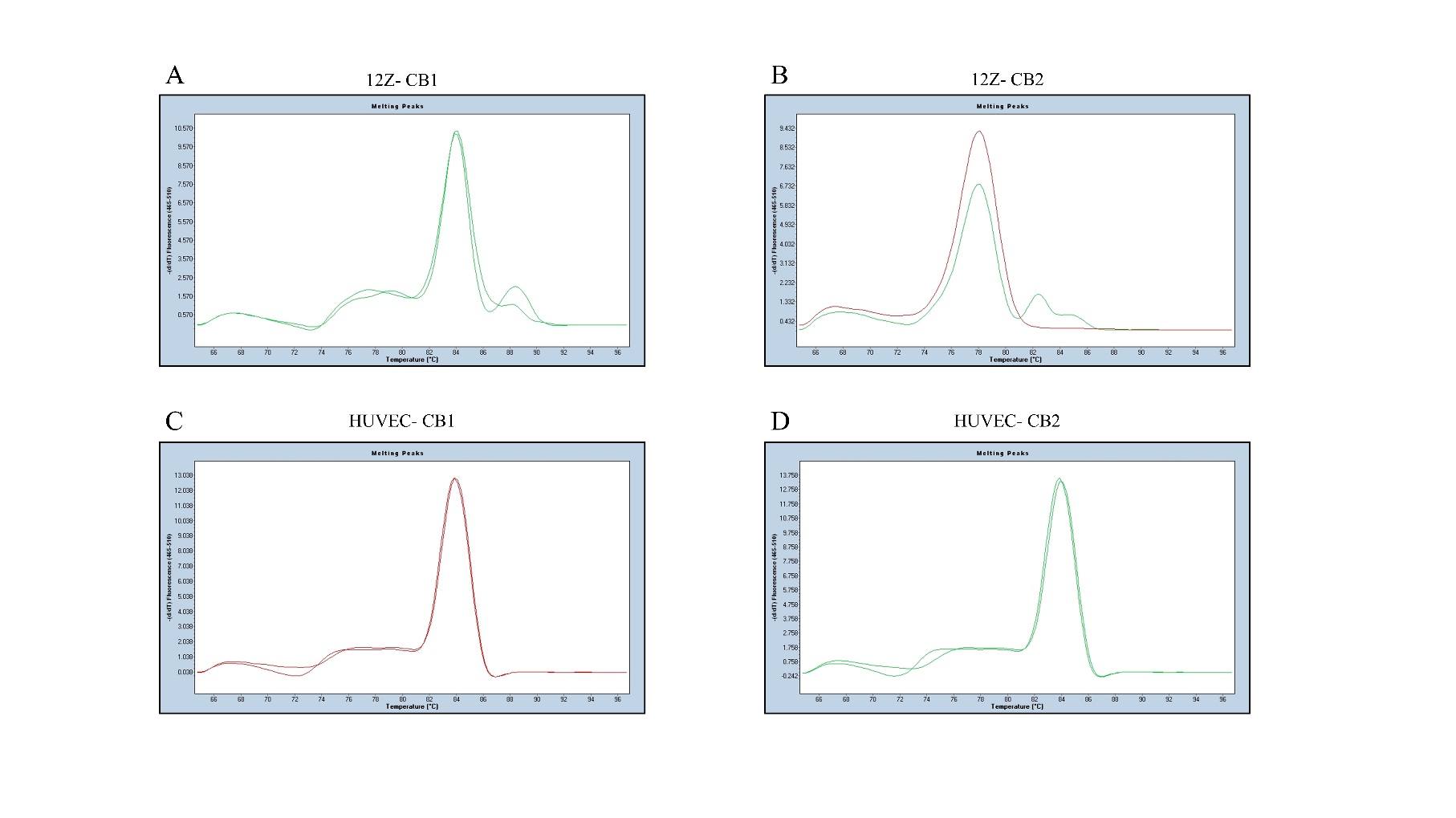
**

**Supplementary Figure 2**


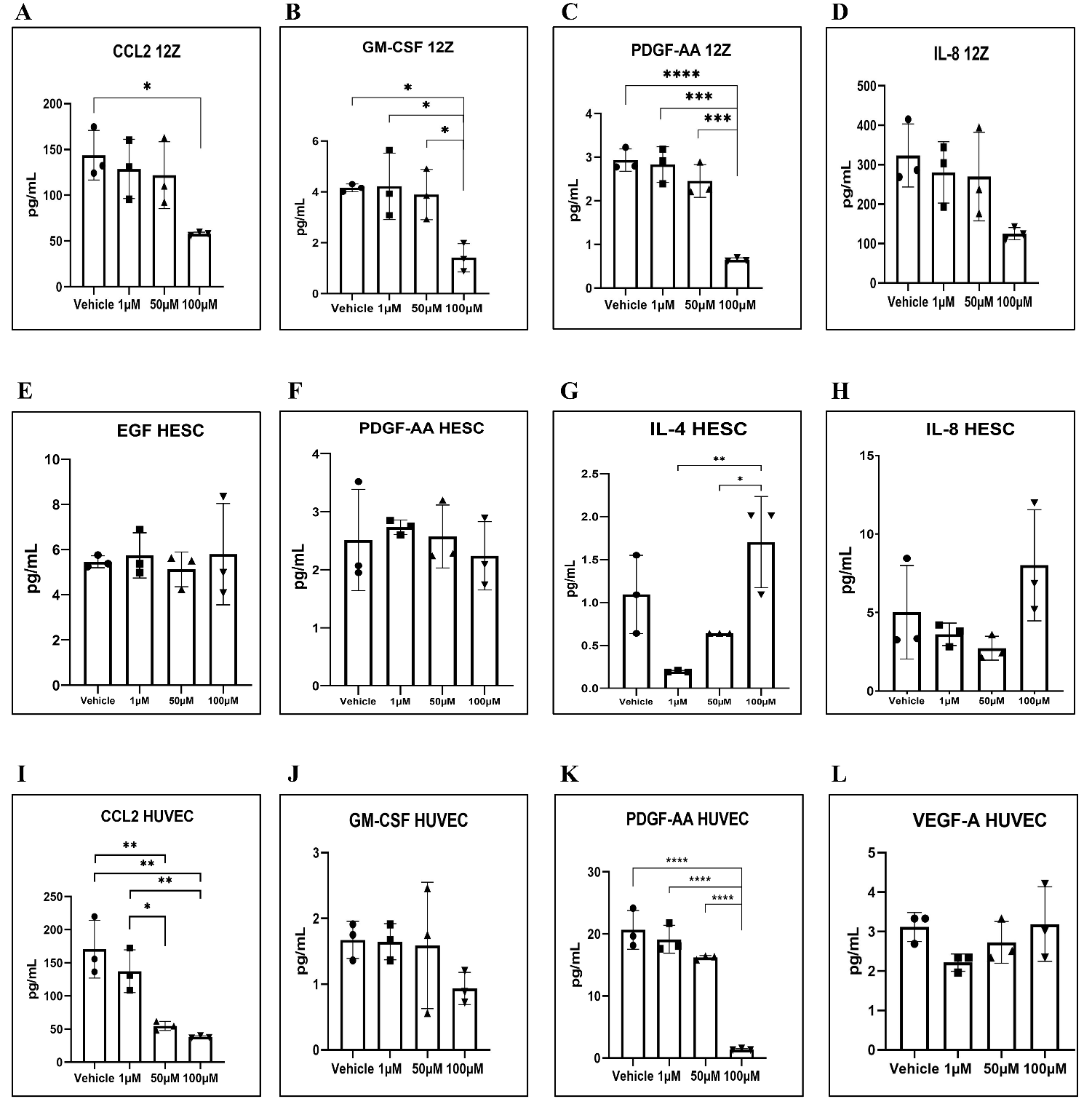


**Supplementary Figure 3**

**
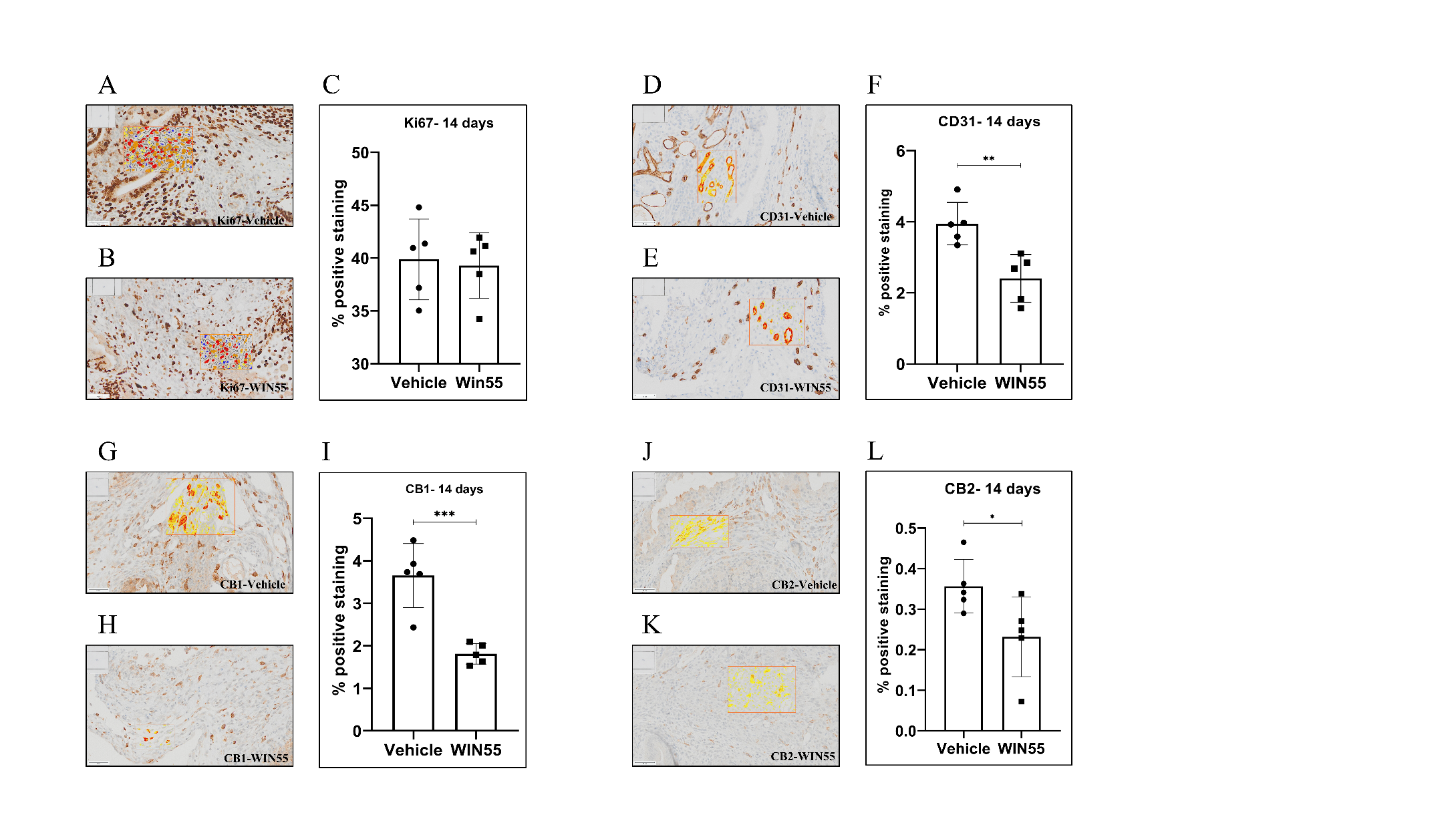
**

**Supplementary Figure 4**

**
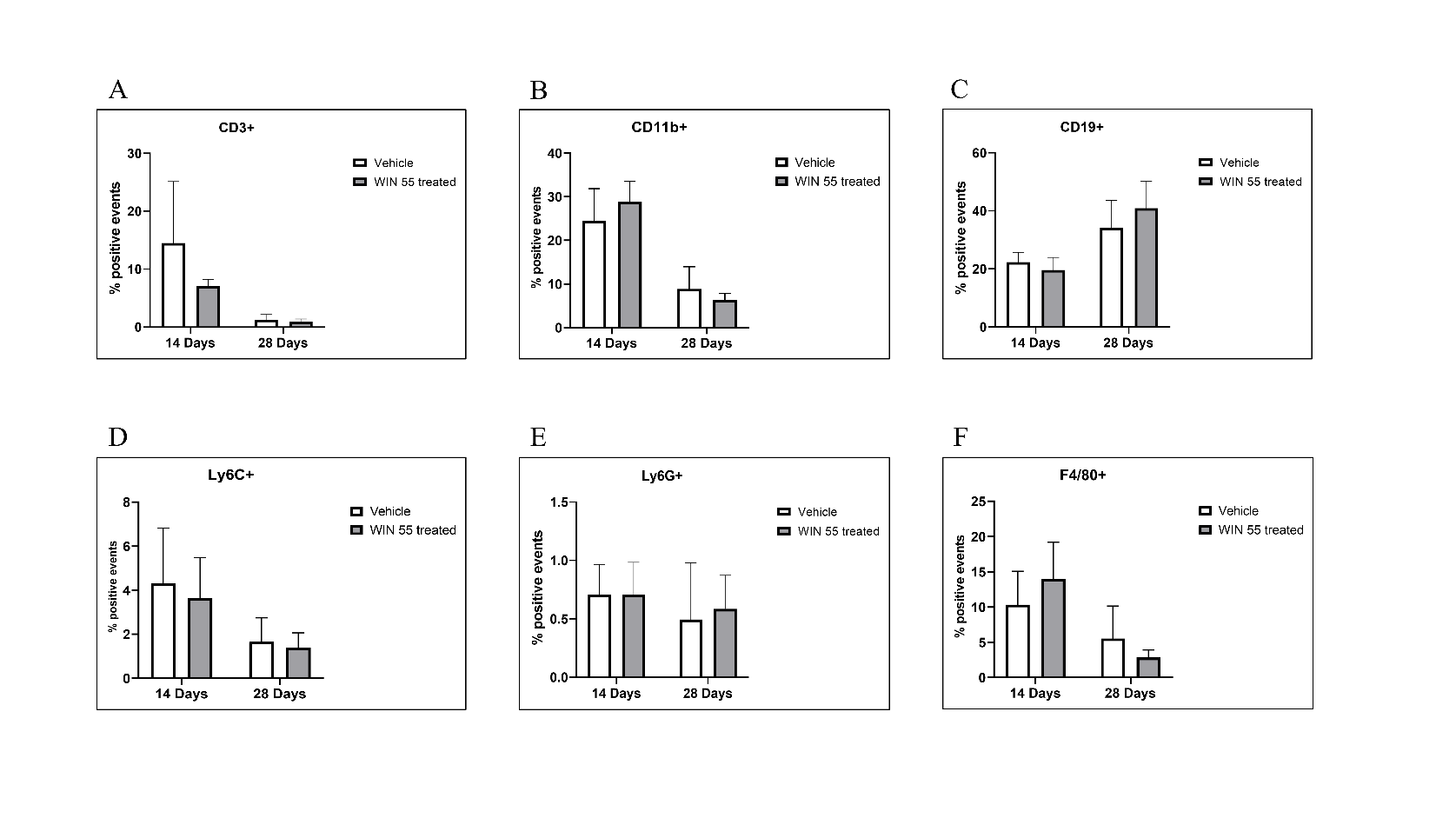
**

**Supplementary Figure 5**

**
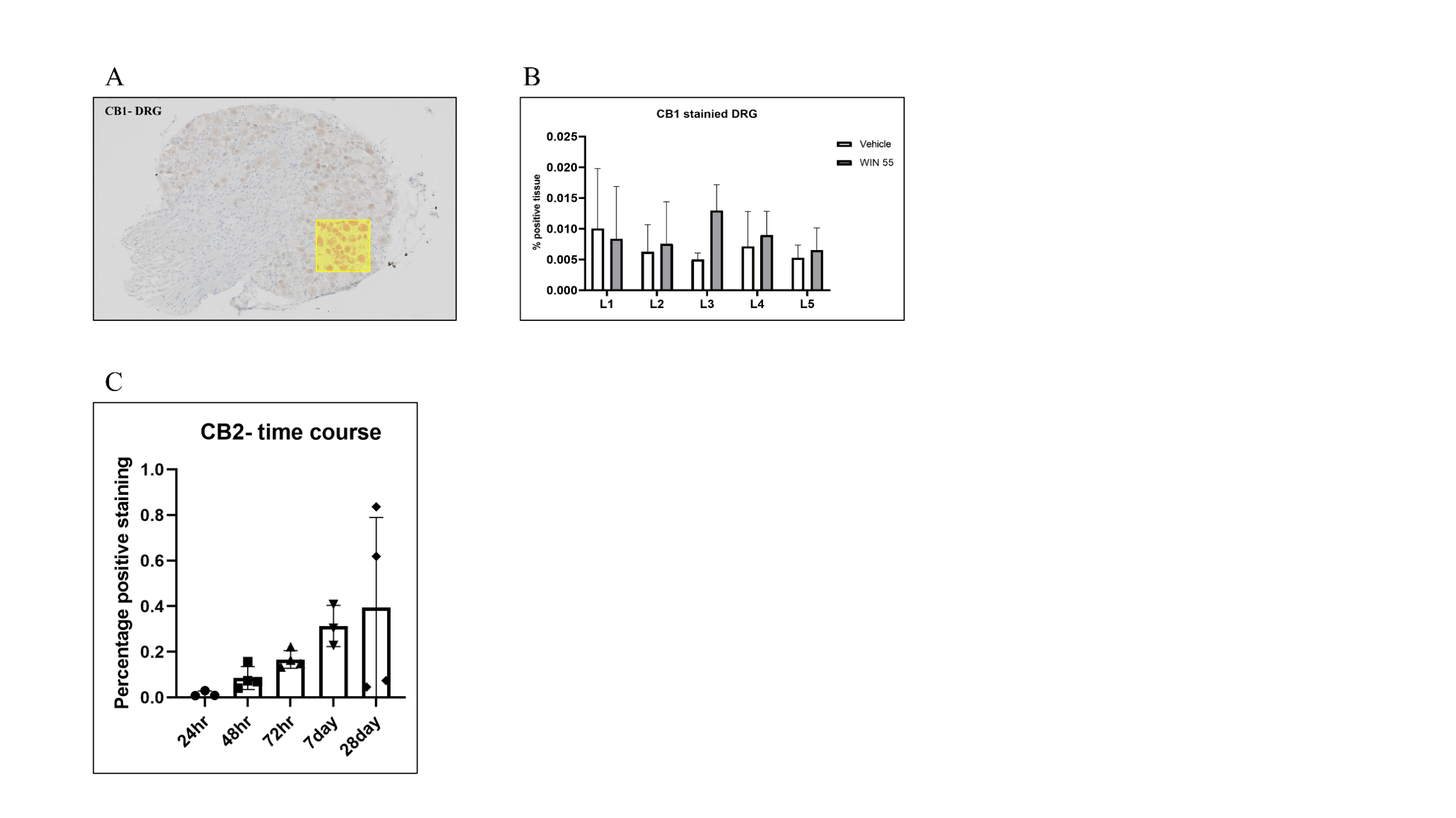
**
